# Effect of tactile and/or kinesthetic stimulation therapy of preterm infants on their parents’ anxiety and depressive symptoms: A systematic review

**DOI:** 10.1186/s40359-023-01510-x

**Published:** 2024-01-02

**Authors:** Cassandre Guittard, Julien Eutrope, Stéphanie Caillies, Gauthier Loron

**Affiliations:** 1 Université de Reims Champagne-Ardenne, C2S, Reims, France; 2 Université de Reims Champagne-Ardenne, C2S, CHU Reims, service de psychiatrie de l’enfant et de l’adolescent, F-51100 Reims, France; 3 Université de Reims Champagne-Ardenne, CReSTIC, CHU Reims, service de médecine néonatale et de réanimation pédiatrique, F-51100 Reims, France

**Keywords:** Preterm, Anxiety, Postnatal depression, Parenting, Proprioception, Skin-to-skin care, Kangaroo care, Tactile and kinesthetic stimulation, NICU

## Abstract

**Background:**

In the case of preterm birth, the idealized postnatal period is replaced by an anxious and even traumatic experience for parents. Higher prevalence of parental anxiety, postnatal depression, and posttraumatic stress disorder has been observed in mothers of preterm infants up to 18 months after childbirth. There is increasing evidence that proprioceptive stimulation has a beneficial effect on preterms’ short-term outcomes. Could this care also have an impact on parental anxiety and depressive symptoms? We reviewed recent publications on the impact on parents’ anxiety and depressive symptoms of delivering tactile and/or kinesthetic stimulation to their premature newborn.

**Methods:**

We conducted a systematic review by searching the PubMed, PsycInfo, Scopus, ScienceDirect and Google Scholar databases for English-language publications from the past 10 years. We focused on the mothers or fathers of infants born preterm (before 37 weeks of gestation) who provided tactile and/or kinesthetic stimulation to their premature newborn in the neonatal intensive care unit. Relevant outcomes were the parents’ anxiety, stress, depressive symptoms, and symptoms of posttraumatic stress disorder, assessed with reliable standardized inventories.

**Results:**

Eleven articles were included in the systematic review. Results suggested a beneficial effect of parents’ early tactile and kinesthetic stimulation of their preterm infants.

**Conclusions:**

These interventions may act as protective factors against the occurrence of anxiety and depressive symptoms in parents and deserve to be studied further in this population.

**Supplementary Information:**

The online version contains supplementary material available at 10.1186/s40359-023-01510-x.

## Background

Preterm birth disrupts early infant development. The technical and stressful environment of the incubator, which separates premature babies from their parents, replaces the natural intrauterine environment. Sleepwake cycles are often interrupted by medical care [1]. The developing brains of premature babies are exposed to stimuli that may be detrimental to their maturation [2], with either too much or too little sensory input [3]. This is referred to as *dystimulation*. Medical comorbidities and sedation impair physiology and makes them less available to interact. On the parental side, the idealized postnatal period is replaced by an anxious and even traumatic experience [4–6]. Parents often express guilt and anxiety about their child’s survival and feel unable to fulfill their parental role [7, 8].

The interaction between the immaturity of the neonate’s organism and this technical environment may affect long-term outcomes, which are consensually reported in the scientific literature for both children and their parents [9–21]. There is evidence that the severity of the infant’s clinical status is related both to the degree of prematurity and to birth weight [12–18]. Multifactorial and diffuse brain damage leads to the loss of vulnerable cells [9–11], but even preterm infants with no overt brain lesions have impaired neurodevelopmental outcomes. These neurodevelopmental problems include impairment of gross and fine motor skills, behavior, and cognition (language, executive functions, and social cognition) [12, 15–18]. In addition, more than 35% of children born prematurely go on to exhibit insecure attachment behaviors in relationships with others [19]. In the long term, these intricate difficulties lead to academic, socio-emotional, relational, and behavioral problems [12, 15–18]. On the parental side, a higher prevalence of parental anxiety, postnatal depression, and posttraumatic stress disorder (PTSD) has been observed in the mothers of preterm infants [20] up to 18 months after childbirth [21]. The parents’ anxiety and depressive symptoms appear to interact with the child’s developmental difficulties (especially in terms of attachment and emotional and cognitive development) in a vicious circle [22, 23].

To avoid these negative consequences of preterm birth, developmental care has been introduced into many neonatal intensive care units (NICUs). Among the most widely studied and practiced forms are the Newborn Individualized Developmental Care and Assessment Program (NIDCAP), the “skin-to-skin care” (SSC) and the “Kangaro mother care” (KMC; which key features are early, continuous skin-to-skin contact between the mother and her baby along with exclusive breastfeeding, ideally [24]). They aim to avoid overstimulation, by protecting the newborn’s most vulnerable sensory systems (e.g., covering the incubator with a blanket, reducing noise levels in the NICU, etc.). They also aim to reduce interruptions of the sleepwake cycle by organizing medical care during the child’s quiet periods of wakefulness and preserving sleep times. Finally, they aim to promote affective contact with parents through the practice of SSC [25]. SSC consists in placing the newborn on the parent’s bare chest. NIDCAP and the SSC have been shown to have many positive effects on the child, such as weight gain, somatic development, maturation of brain electrical activity as measured by EEG, fewer medical complications, a shorter hospital stay, and long-term cognitive development [26], as well as on maternal wellbeing [27, 28].

Several teams are currently testing whether targeted enhancement of preterm infants’ sensory environment has an additional positive effect, compared with the usual developmental care. The common purpose is to provide stimulation to promote physiological maturation of neuronal networks sensitive to (or dependent on) sensory input. One challenge is to avoid dystimulation by compensating for under-stimulation in the tactile, kinesthetic or vestibular sensory modalities while protecting the visual and auditory modalities from over-stimulation. Some authors have investigated the potential of massage care, which consists of touch with gentle pressure. Several studies have shown that massage care in very preterm infants is associated with reduced stress levels, less late sepsis, improved hemodynamic stability, increased weight gain, and enhanced parental wellbeing [29–32]. Other studies have focused on the effects of tactile and kinesthetic stimulation (TKS) on preterm infants. TKS is a combination of moderate tactile stimulation and kinesthetic stimulation through flexion and extension movements of the limbs. It has been shown to have many beneficial effects on physiological measures (e.g., heart rate, state of alertness, breastfeeding) [33, 34], length of hospital stay [33], maturation of brain electrical activity measured by EEG [35], early interactions and attachment [36], and neurodevelopmental outcomes [36–38].

These studies underline the relevance of these types of stimulation for premature children. But what about the parents who provide this stimulation? As we mentioned earlier, preterm mothers have been found to have higher prevalence of parental anxiety, postnatal depression, and PTSD [20] up to 18 months after childbirth [21]. The main purpose of this systematic review was to highlight the impact on parents’ anxiety and depressive symptoms of providing stimulation (i.e., SSC and TKS) to their premature newborn.

There is a consensus that children’s neurodevelopmental outcomes are the result of interaction between their genetic and neurobiological phenotype and their environment [39–41], which includes their relationship with their parents. Parents’ behaviors toward their children therefore affect their development [42, 43]. Parents’ ability to interact with their children has been shown to be modified by anxiety and depressive symptoms [44, 45]. When interacting with their young children, parents with these disorders oscillate between moments of withdrawal and moments of intrusion [44, 45]. These atypical early interactions affect children’s cognitive, social and emotional development [46–50]. In very young children, sleep and feeding difficulties have been reported [51]. In extreme cases, if parents’ mental health difficulties are left untreated, they can become a risk factor for child neglect and even abuse [52], especially when the child has specific needs (e.g., prematurity or disability) [53]. It is therefore relevant to ask whether the care given to premature infants could also be beneficial for their parents, if they are the ones who provide it. The present systematic review investigated this question by focusing on the impact on parents’ anxiety and depressive symptoms of providing tactile and/or kinesthetic stimulation to their premature newborn.

## Methods

### Objective

We used the PopulationInterventionComparisonOutcomes (PICO) method to specify the components of our systematic review’s main research question. The population (P) was the parents of premature newborns, the intervention (I) was tactile and/or kinesthetic stimulation delivered by parents to their premature newborn, in comparison (C) with standard care in the NICU (including developmental care), and the outcomes (O) were parental anxiety and depressive symptoms.

### Procedure

#### Search strategy

We searched the PubMed, PsycInfo, Scopus, ScienceDirect and Google Scholar databases, using the following keywords: (preterm AND (kangaroo care OR skin-to-skin OR massage OR tactile OR proprioceptive OR kinesthetic) AND (anxiety OR depression OR depressive OR traumatic OR stress)). Additional terms were associated with these keywords (e.g., for preterm: premature, prematurity). We restricted the search to English-language articles published within the previous 10 years (i.e., between January 2012 and December 2022). We also searched the reference lists of included studies for additional eligible articles.

### Inclusion and exclusion criteria

We looked for studies that focused on the mothers or fathers of infants born prematurely (before 37 weeks of amenorrhea) who provided tactile and/or kinesthetic stimulation to their premature newborn in NICU. Relevant outcomes were the parents’ anxiety, stress, depressive symptoms, or symptoms of PTSD, as assessed with reliable standardized inventories. Articles in which parents performed additional stimulation in another sensory modality (e.g., auditory stimulation through music) were excluded. Studies assessing symptoms without the use of reliable standardized inventories were also excluded.

### Study selection and quality assessment

We used the PRISMA method to select the articles. An initial selection was made by the first author according to the title, and a second selection was based on the abstract. A final selection was made after a full-text reading of each article and analysis of its quality using the Joanna Briggs Inventory checklists. These checklists assess the quality of articles according to the following criteria: randomization, blinding of participants and assessors, treatment groups, follow-up of participants, methods of measuring outcomes (tools, time, etc.), and statistical analyses. Each criterion was rated as either met (*yes*), not met (*no*), not clearly met (*unclear*), or not applicable. It should be noted that the participant blinding criterion could not be applied here, given that the stimulation was provided by the participants themselves. The percentage risk of bias was therefore calculated on the remaining items (i.e., 10 items out of 13 for the Checklist for Randomized Controlled Trials, and 10 items out of 11 for the Checklist for Cohort Studies). A high risk of bias corresponds to a score between 0% and 50%, a moderate risk to a score between 51% and 70%, and a low risk to a score between 71% and 100%. A percentage risk of bias was calculated for each article (see Appendix 1).

**Fig. 1:**
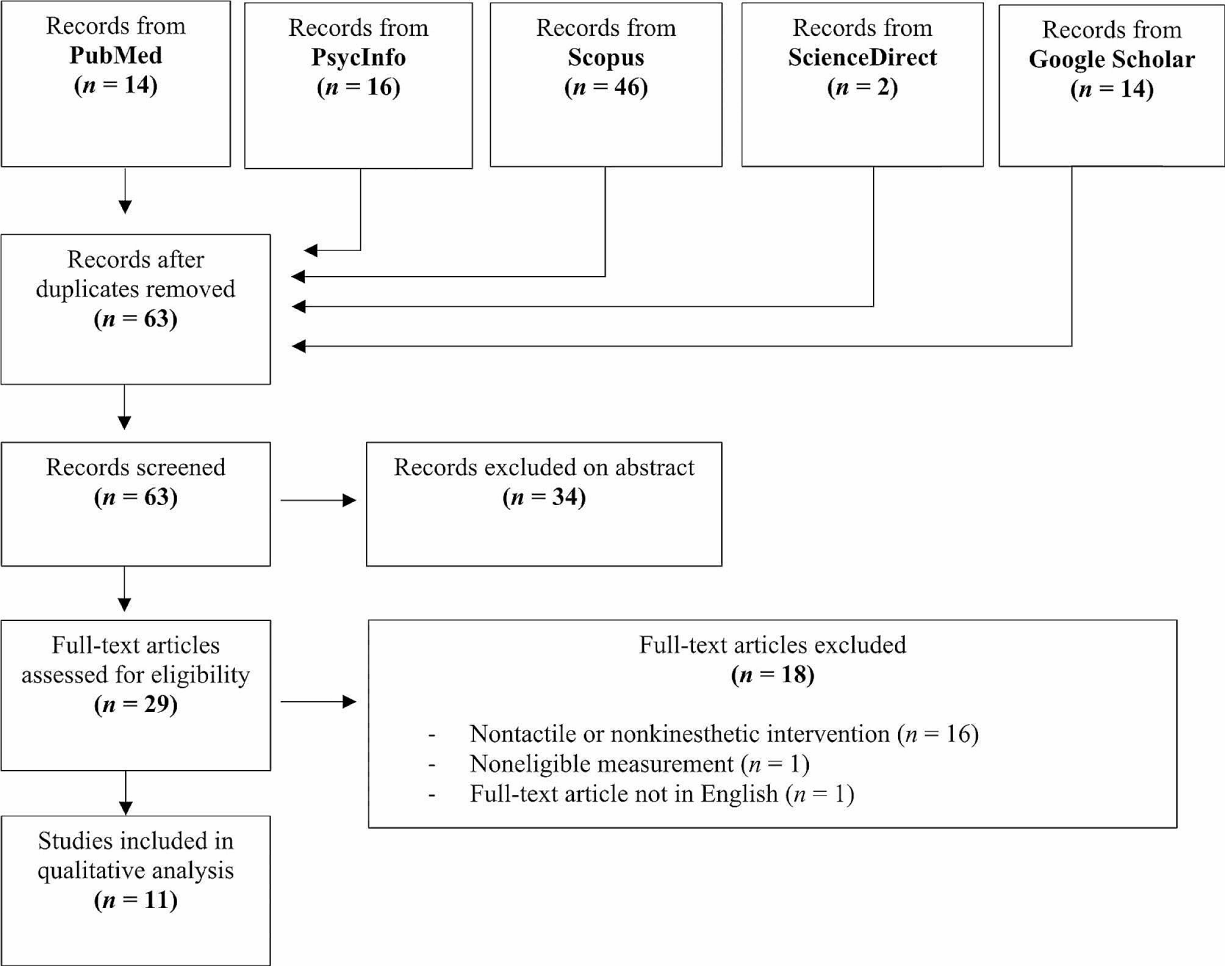
Flow diagram

The first author helped by a master student performed the selection and qualitative assessment of the articles. In case of discrepancy about selected/rejected articles, the whole team assessed the articles under consideration and built consensus whether or not each article should be retained. The article selection steps are detailed in Fig. 1.

### Data extraction

The data extracted from the articles regarding the population, stimulation (nature, duration, frequency, etc.), measurement tools, measured outcomes, and statistical results were compiled independently by the first author and the master student. Their respective extractions were then compared by the team to fill in any missing data. Given the heterogeneity of the methodologies and results in the selected articles, we undertook a qualitative synthesis of the data.

## Results

Of the 309 studies we screened, 63 were assessed for eligibility. A total of 34 articles were excluded after reading the abstract. A further 18 articles were excluded after reading the full text for the following reasons: (a) 16 articles dealt with an ineligible intervention (i.e., nontactile and/or nonkinesthetic stimulation); (b) one article did not measure parental anxiety and depressive symptoms with reliable standardized inventories; and (c) one article was not in English. Accordingly, 11 studies met our inclusion criteria and were included in this systematic review [27, 28, 31, 32, 36, 38, 54–58].

Two articles reported the effect of KMC [56, 57], four focused on the effect of SSC [27, 28, 54, 57], three on the effect of TKS [36, 38, 58], and two compared the effects of SSC and TKS [32, 33].

The studies we selected involved infants born between 25 and 37 weeks of gestation. All these studies included late preterm infants (33–37 weeks), eight studies included very preterm infants (26–32 weeks) and six studies included extremely preterm infants (< 26 weeks). These data are summarized in Table 1. After examining the respective effects of SSC, KMC and TKS on parents’ anxiety, stress, depressive symptoms, and symptoms of PTSD, we report the comparative effects of SSC versus TKS.

**Table 1:** Characteristics of studies included in systematic review

Authors year	Sample and population	Intervention	Control group	Outcome measurements	Primary endpoint	Timing of assessment	Main findings on parental symptoms
Afand et al. (2021)	*N* = 70 32–37 wGA, only mothers included	Massage with almond oil “(1) from the head down to the neck and vice versa, (2) from the upper back down to the waist, and vice versa” for 8 min once a day for 2 days: 4 min by the mother, and 4 min by a healthcare professional.	Standard medical care only. No massage.	Maternal anxiety: State-Trait Anxiety Inventory (STAI)	STAI	Longitudinal measurement in both groups: day before discharge (T1, before intervention for the experimental group) and discharge day (T2)	STAI scores decreased significantly between T1 and T2 in both groups. At T2, the massage group’s STAI scores were significantly lower than those of the control group.
Erduran et al. [56]	*N* = 60, < 37 + 6 wGA, only mothers included	Kangaroo care 30 min per day for 10 days.	Standard medical care only. No kangaroo care.	Edinburgh Postnatal Depression Scale (EPDS) Maternal Attachment Inventory (MAI)	MAI	30–40 days after birth	The EPDS scores of the kangaroo care group were lower than those of the control group, but the difference was not statistically significant.
Feldman et al. [54]	*N* = 146, 25–34 wGA, only mothers included	Skin-to-skin care 1 h per day for 14 days.	Standard medical care only. No skin-to-skin care.	Physiological measures: heart rate, sleep, cortisol level Cognitive measures: Bayley Scale of Infant Development, Wechsler Intelligence Scale for Children (WISC), and NEPSY Measurement of early interactions: Coding Interactive Behavior (CIB) Measures of maternal anxiety and depressive symptoms: STAI, EPDS, Parental Stress Index (PSI)	Not specified	At 3 months, 6 months, and 10 years (corrected age)	The STAI and PSI scores of the skin-to-skin group were significantly lower at 3 months and 6 months (corrected age). This difference was no longer significant at 10 years. There was no significant difference in BDI scores between the two groups.
Gholami et al. [31]	*N* = 90, 28–36 wGA, only mothers included	Two intervention groups: 1. Massage: Tactile stimulation by moderate touching of the head, neck, shoulders, back and lower limbs. Kinesthetic stimulation by arm and hand movements for 5 min 3 times a day until discharge. 2. Kangaroo care for between 20 min and 3 h 3 times a day until discharge.	Standard medical care only. No massage or kangaroo care.	Neonatal Infant Pain Scale (NIPS) STAI	STAI	Longitudinal measurements at hospitalization (T1, before intervention for massage and skin-to-skin groups) and at discharge (T2, after intervention)	The STAI scores of the massage group were lower than those of the kangaroo care group, but this difference was not statistically significant. The STAI scores of the massage and skin-to-skin groups were significantly lower than those of the control group.
Herizchi et al. [27]	*N* = 60, < 37 wGA, only mothers included	More than 3 h of skin-to-skin care daily for 1 month.	Less than 3 h of skin-to-skin care per day for 1 month.	EPDS	EPDS	10, 20 and 30 days after childbirth	No significant difference between groups at D10. The EPDS scores of the experimental group were significantly lower than those of the control group at D20 and D30. Between D10 and D30, the EPDS scores of the experimental group decreased, while the scores of the control group increased.
Karimi et al. [32]	*N* = 90, 28–36 + 6 wGA, only mothers included	Two intervention groups: 1. Massage: “with the infants in a prone position, applying moderate pressure stroking to the head, shoulders, back, legs and arms for 5 minutes, (2) kinesthetic stimulation consisting of flexing and extending the limbs in a supine position for the next 5 minutes, and (3) returning the infant to the prone position and repeating the moderate pressure massage stroking sequence for the last 5 min (same as the first 5 minutes).” 3 times a day for 5 days. 2. 15 min kangaroo mother care twice a day for 5 days.	Standard medical care only. No massage or kangaroo mother care.	Depression, Anxiety and Stress Scale (DASS-21) NIPS Coping Responses Inventory for Adults (CRI-A)	DASS-21	Longitudinal measurements before and 5 days after the interventions for each group	Levels of anxiety, stress, and depressive symptoms were significantly lower in the massage and kangaroo mother care groups than in the control group after the interventions. There was no significant difference between the massage and kangaroo mother care groups.
Mokaberian et al. (2021)	*N* = 40, 32–37 wGA, only mothers included	Massage with 8 min of tactile stimulation + 4 min of kinesthetic stimulation + 8 min of tactile stimulation 3 times a day for 10 days.	Standard medical care only. No massage.	Neonatal Behavioural Assessment Scale (NABS) Peabody Development Motor Scale (PDMS) Maternal Postnatal Attachment Scale (MPAS) STAI	Not specified	Not specified	The STAI scores of the massage group were significantly lower than those of the control group.
Mörelius et al. [57]	*N* = 42, 32–35 + 6 wGA, mothers and fathers included	Continuous skin-to-skin care until discharge.	Unrestricted skin-to-skin care (i.e., as many times as the parent wished).	Physiological measurement: cortisol level Measurement of parental anxiety and depressive symptoms: Swedish Parenthood Stress Questionnaire (SPSQ) EPDS Interview including questions about breastfeeding	SPSQ and EPDS	At 1 month and 4 months (CA)	No significant difference between groups on SPSQ scores. The EPDS scores of the continuous skin-to-skin group were lower than those of the unrestricted skin-to-skin group for the mothers, but this difference was not significant.
Ochandorena-Acha et al. [58]	*N* = 48, 28–34 wGA, only mothers included	Between 32 and 36 wGA: 10 min of tactile stimulation with moderate pressure + 5 min of kinesthetic stimulation by movements of the upper and lower limbs. Twice a day for 10 days. Between 40 wGA and 2 months (CA): positions and movements facilitating motor development and exploration of objects, with 4 home supervision visits. 15–20 min twice a day for 5 days a week.	Newborn Individualized Developmental Care and Assessment Program (NIDCAP)	Alberta Infant Motor Scale (AIMS) Ages and Stages Questionnaires Third Edition (ASQ-3) Parenting Stress Index-Short Form (PSI-SF)	AIMS	AIMS at 2 and 8 months (CA), ASQ-3 at 1 month (CA), PSI-SF at 3 months (CA)	No significant difference between the groups.
Rao et al. [55]	*N* = 50, < 37 wGA, only mothers included	Kangaroo mother care minimum 4 h per day for 1 week.	No control group.	Hospital Anxiety and Depression Scale (HADS)	HADS	Longitudinal measurement at D0 (T1, before intervention) and D7 (T2, after intervention)	Levels of anxiety and depressive symptoms were significantly lower after the intervention.
Sweeney et al. [28]	*N* = 116, < 34 wGA, mothers and fathers included	Skin-to-skin care for 30 min twice a day.	No control group.	STAI	STAI	Longitudinal measurement before and after intervention (minimum 1 h of skin-to-skin care)	STAI scores were significantly lower after the intervention.

*Note*: wGA = weeks gestational age; CA = chronological age

Parental symptoms were assessed with a number of standardized tools. *Anxiety* was assessed with the StateTrait Anxiety Inventory [59] in five articles [28, 31, 36, 54, 58], the Hospital Anxiety and Depression Scale (HADS) [60] in one article [55], and the Depression Anxiety Stress Scale (DASS-21) [61] in one article [32]. *Parental stress* was assessed with the Parenting Stress Index in two articles [38, 54], the Swedish Parenthood Stress Questionnaire in one article [57], and the DASS-21 in one article [31]. *Depressive symptoms* were assessed with the Edinburgh Postnatal Depression Scale [62] in three articles [27, 56, 57], the Beck Depression Inventory [63] in one article [54], the HADS in one article [55], and the DASS-21 in one article [32]. *PTSD* was not assessed in any of the articles.

### Effects of skin-to-skin care on parental anxiety and depressive symptoms

Two trials consistently showed an association between SSC and a reduction in maternal anxiety was consistently demonstrated in two studies. In these studies, SSC was performed one hour per day for periods ranging from 1 to 14 days. Sweeney et al. [28] demonstrated a significant reduction in maternal anxiety after practicing SSC [27]. Feldman et al. [54] reported lower maternal anxiety at 3 and 6 months postpartum among mothers of babies born between 25 and 34 weeks who had practiced SSC in NICU, but this difference was no longer significant of age 10 years [54].

Only two studies assessed the effect of SSC on parental stress, and their results were divergent. Feldman et al. [54] reported lower parental stress at 3 and 6 months postpartum among mothers who had practiced SSC in NICU, but this difference was no longer significant at 10 years [54]. Mörelius et al. [57] found no significant effect of SSC on parental stress among either mothers or fathers [57].

Two studies found a beneficial effect of SSC on parents’ depressive symptoms. One study reported lower depressive scores in the first month postpartum in mothers who practiced SSC [27]. Mörelius et al. [57] showed lower scores of depressive symptoms in mothers who practiced continuous skin-to-skin (i.e., 24 h per day, 7 days per week) than in mothers who practiced standard SSC (i.e., as many times as the parent wished), but this effect did not reach statistical significance [57].

### Effects of kangaroo mother care on parental anxiety and depressive symptoms

Two studies reported the effect of KMC (skin-to-skin contact combined with exclusive breastfeeding) on parental and/or depressive symptoms. However, it is important to note that in these trials, skin-to-skin contact was not practiced continuously, contrary to the recommended methodology in the KMC. Indeed, in these studies, SSC was performed between one and four hours per day for one week. Erduran et al. [56] thus refers to this as “intermittent kangaroo care”.

Only the Rao et al.’s study [55] reported the effect of KMC on parental anxiety. It showed a decrease in parental anxiety symptoms during the first week postpartum after performing KMC [55].

Both studies found a beneficial effect of KMC. Rao et al. [55] showed a significant decrease in depressive symptoms during the first week postpartum after performing KMC [55] and Erduran et al. [56] observed fewer depressive symptoms during the first month postpartum in parents who practiced KMC, although this effect did not reach statistical significance [56].

### Effects of tactile and kinesthetic stimulation on parental anxiety and depressive symptoms

The effect of TKS on maternal anxiety was investigated in two studies. In these studies, TKS was performed for at least 8 min per day (maximum of 45 min per day), for 2–60 days). Both studies found that mothers who practiced TKS had less anxiety than those who did not, from the first week after childbirth [36, 58].

A single study investigated the effect of TKS on maternal stress [38]. This study reported no significant difference in stress at 3 months postpartum between mothers of infants born after 28–34 weeks of gestation who practiced TKS and those who did not.

None of the studies investigated the effect of TKS on parents’ depressive symptoms.

### Effects of skin-to-skin contact versus tactile and kinesthetic stimulation on parental anxiety and depressive symptoms

Two articles compared the effects of SSC versus TKS on parental anxiety and depressive symptoms, yielding divergent results [31, 32]. The first study compared the anxiety levels of mothers of infants born after 28–36 weeks of gestation who received either SSC, TKS, or standard care (control group) [31]. Mothers in the SSC and TKS groups had significantly lower anxiety scores than those in the control group, but there was no significant difference between mothers in the SSC versus TKS groups. The second study compared the effects of SSC and TKS on the anxiety, stress, and depressive symptoms of mothers of infants with a gestational age of 28–36 weeks [32]. In this study, mothers who performed SSC had significantly lower levels of anxiety, stress, and depressive symptoms than mothers who performed TKS.

## Discussion

The purpose of this systematic review was to highlight recent findings on the effects on parents’ anxiety and depressive symptoms of providing tactile and/or kinesthetic stimulation to their premature newborn. To address this issue, we screened the results of 11 articles dealing with tactile sensory interventions, including four articles on the effects of SSC, two on the effects of KMC, three on the effects of TKS, and two articles comparing the effects of SSC and TKS.

Results were unequivocal for the beneficial effects of sensory stimulation on maternal anxiety, with a significant decrease in symptoms from the first week of practice, whether the stimulation was solely tactile (SSC) or tactile and kinesthetic (TKS). Results were more mixed for parental stress, as most studies failed to find significant effects of either SSC or TKS. Regarding depressive symptoms, studies of SSC only found a trend toward a significant effect. It should be recalled that none of the studies specifically investigated the effect of TKS on depressive symptoms, and the comparison between TKS and SSC in one study failed to yield conclusive results. Moreover, none of the studies investigated the effects of either SSC or TKS on the symptoms of PTSD. There is therefore a dearth of scientific research on the effects of SSC and/or TKS on depressive symptoms and the symptoms of PTSD. Given that depressive symptoms in the postnatal period have been identified as being predictive of PTSD symptoms [64], it would be even more relevant to assess all the components of parental anxiety and depressive symptoms in relation to sensory interventions. Similarly, only two studies compared the effects of SSC and TKS. The heterogeneity of their methods and their divergent results did not allow us to draw any conclusions regarding the differential effects of TKS versus SSC. Moreover, as SSC has become standard care in NICUs, it would be more ecological for future studies to investigate the additional effect of TKS when associated with SSC [65].

The authors provided different explanations for the beneficial effects of SSC and TKS on parental anxiety and depressive symptoms. One of these focused on the physiological benefits of tactile and kinesthetic contact. Maternal oxytocin production has been found to be disturbed after premature birth [54, 66], and more generally in parents with anxiety and depressive symptoms [67–70]. However, tactile and affectionate contacts increase plasma levels of oxytocin and endorphins in both infants and their parents [71–73]. These hormones have been shown to improve parents’ mood by reducing their cortisol levels (associated with stress) [74–77] and giving them feelings of security, wellbeing, and happiness [77, 78]. The second explanation took more account of the social environment and the psychological mechanisms involved. As the parentinfant interaction is disrupted, the parents of premature infants often express a feeling of parental incompetence [79–81] characterized by the feeling of not being useful in caring for their baby (as most newborn care is performed by professionals and not by the parents themselves), and not being able to understand or respond to their baby’s needs. This contributes to the emergence of anxiety, depressive symptoms, and feelings of powerlessness that heighten the traumatic nature of hospitalization in NICU [4, 82, 83]. Practicing SSC or TKS places parents at the center of their infant’s care, giving them an important role. The daily benefits that they can see in their child’s condition reduce their feeling of parental incompetence [84]. By acting at both physiological and psychological levels, TKS is likely to have a dual effect. SSC and TKS may therefore act as protective factors against the occurrence of parental anxiety and depressive symptoms.

Further studies are needed to confirm the beneficial effects of SSC and/or TKS on parents’ depressive symptoms and symptoms of PTSD. As indicated earlier, studies have not always yielded significant statistical results. These divergent results can be explained by the heterogeneity of the interventions, in terms of the frequency and duration of stimulation. The different methods used to assess anxiety and depressive symptoms, in terms of tools and timing, may be another explanation. Finally, the discrepancy in results could also be explained by the diversity of the samples. The studies included in this review were conducted among the parents of preterm babies of different gestational ages. The parents and their babies therefore faced different risk factors, newborn health conditions, and hospitalization durations, which may have had differential effects on their anxiety and depressive symptoms. Maternal anxiety and depressive symptoms have been shown to correlate with the infant’s gestational age [21, 85–87], medical risk factors such as birth weight [86–88], neonatal risk [48, 51, 86, 87, 89], cesarean Sect. [21] and medical complications [85, 88, 89], and length of hospital stay [90]. Sensory interventions may therefore need to be longer to have a statistically significant beneficial effect in parents, if the neonatal risk is particularly high. In addition, most studies only included mothers in the intervention protocol, whether it be for the practice of sensory stimulation or the assessment of anxiety and depressive symptoms. However, fathers also form part of the child’s daily relational environment, and recent studies have shown that anxiety and depressive symptoms are also present in the fathers of preterm infants [91, 92], and therefore have an impact on these infants’ development [93–97]. Accordingly, it is important to include fathers in future studies and to develop scales to assess perinatal anxiety and depressive symptoms in fathers.

There can no longer be any doubt about the importance of developing interventions to reduce parental anxiety and depressive symptoms, given that there is so much at stake. Maternal anxiety and depressive symptoms are one of the main causes of postpartum suicide [98], and a risk factor for child neglect or abuse [99]. Moreover, parental anxiety and depressive symptoms have been shown to adversely affect sleep quality and feeding in early life [51], and cognitive, affective, social, and behavioral development in later childhood, even outside the context of prematurity [44, 48, 100–104]. Studies supporting the diathesisstress theory, according to which early exposure to stressors makes individuals more sensitive and receptive to negative influences from their subsequent environment, have shown that parental anxiety and depressive symptomatology has an even more detrimental effect on child development when it occurs in a context that is already a source of vulnerability, as in the case of preterm birth [48, 105, 106]. Researchers and clinicians need to work together to develop such interventions to reduce anxiety and depressive symptoms in the parents of preterm infants.

### Limitations

Our systematic review had three main limitations. First, the heterogeneity of the infants’ gestational age in the included studies prevented us from specifying the effects of tactile and/or kinesthetic stimulation according to the stage of prematurity. This is nevertheless a promising avenue for research, given that researchers have demonstrated a correlation between parental symptoms and gestational age [21, 107, 108], and the benefits of stimulation increase with the severity of prematurity [109–111].

Second, some of the studies did not provide enough details about the characteristics of their sample (e.g., gestational age), the timing of the assessment of parental anxiety and depressive symptoms, and the sensory stimulation protocol. The validation of inventories translated into different languages and cultures might make it easier to reproduce these studies. Similarly, detailed descriptions of the stimulation provided to infants born preterm are mandatory for further replication studies.

Third, we selected studies published in English within the previous 10 years to examine the results of the most recent international scientific literature. However, there are certainly older studies and/or studies published in other languages that would provide interesting results.

## Conclusion

In conclusion, providing early tactile and kinesthetic stimulation to their preterm infants seems to have a beneficial effect on parents’ anxiety and depressive symptoms. These interventions protect against the occurrence of symptoms and could be used for prevention in at-risk populations.

### Electronic supplementary material

Below is the link to the electronic supplementary material.

Supplementary Material 1

## Data Availability

Not applicable.

## Abbreviations

PTSD: posttraumatic stress disorder
NICU: neonatal intensive care unit
NIDCAP: Newborn Individualized Developmental Care and Assessment Program
SSC: skin-to-skin care
TKS: tactile and kinesthetic stimulation
PICO: Population–Intervention–Comparison–Outcomes
HADS: Hospital Anxiety and Depression Scale
DASS-21: Depression Anxiety Stress Scale
