# Supplementary material for: Effect of tactile and/or kinesthetic stimulation therapy of preterm infants on their parents’ anxiety and depressive symptoms: A systematic review
Source: BMC Psychol. 2024 Jan 2;12:3. doi: 10.1186/s40359-023-01510-x (PMC10759426; doi:10.1186/s40359-023-01510-x)
Supplement: Supplementary file 1 — Supplementary Material 1 [file 40359_2023_1510_MOESM1_ESM.docx]

**Appendix 1. Table summarizing Joanna Briggs Inventory** (**JBI) risk of bias assessment of articles included in the systematic review**

| **Authors**  **Year** | **Risk severity** | **Risk of bias** | **Biases identified** |
| --- | --- | --- | --- |
| Checklist for Randomized Controlled Trials | | | |
| Afand et al., 2017 | Moderate | 70% | 1. Was true randomization used for assignment of participants to treatment groups? *(No)*  6. Were outcomes assessors blind to treatment assignment? *(Unclear)*  13. Was the trial design appropriate, and any deviations from the standard RCT design (individual randomization, parallel groups) accounted for in the conduct and analysis of the trial? *(Unclear)* |
| Erduran et al., 2022 | Low | 100% |  |
| Feldman et al., 2014 | Low | 100% |  |
| Gholami et al. 2021 | Low | 90% | 8. Was follow up complete and if not, were differences between groups in terms of their follow up adequately described and analyzed? *(Unclear)* |
| Herizchi et al., 2017 | Moderate | 70% | 1. Was true randomization used for assignment of participants to treatment groups? *(No)*  3. Were treatment groups similar at the baseline? *(Unclear)*  6. Were outcomes assessors blind to treatment assignment? *(Unclear)* |
| Karimi et al. 2021 | Low | 90% | 8. Was follow up complete and if not, were differences between groups in terms of their follow up adequately described and analyzed? *(Unclear)* |
| Mokaberian et al., 2021 | Low | 90% | 6. Were outcomes assessors blind to treatment assignment? *(Unclear)* |
| Mörelius et al., 2015 | Low | 100% |  |
| Ochandorena-Acha et al., 2022 | Low | 100% |  |
| Checklist for Cohort Studies | | | |
| Rao et al., 2019 | Low | 90% | 8. Was the follow up time reported and sufficient to be long enough for outcomes to occur? *(No)* |
| Sweeney et al., 2017 | Moderate | 70% | 5. Were strategies to deal with confounding factors stated? *(No)*  8. Was the follow up time reported and sufficient to be long enough for outcomes to occur? *(No)*  10. Were strategies to address incomplete follow up utilized? *(Unclear)* |
